# CD30-Positive Anaplastic Variant of Diffuse Large B-cell Lymphoma: Frequency and Association With Clinicopathological Parameters

**DOI:** 10.7759/cureus.13209

**Published:** 2021-02-07

**Authors:** Atif A Hashmi, Rimsha Haider, Gul Nargus, Omer Ahmed, Syed Rafay Yaqeen, Ishaq Azeem Asghar, Anoshia Afzal, Muhammad Irfan, Muhammad M. Edhi, Javaria Ali

**Affiliations:** 1 Pathology, Liaquat National Hospital and Medical College, Karachi, PAK; 2 Internal Medicine, Liaquat National Hospital and Medical College, Karachi, PAK; 3 Emergency Medicine, National institute of Blood Diseases and Bone Marrow Transplantation, Karachi, PAK; 4 Pathology, Khyber Medical college, Peshawar, PAK; 5 Internal Medicine, Baqai Medical University, Karachi, PAK; 6 Pathology, Ascension St. John Hospital, Detroit, USA; 7 Pathology, University of Oklahoma Health Sciences Center, Oklahoma City, USA; 8 Statistics, Liaquat National Hospital and Medical College, Karachi, PAK; 9 Neuroscience/Neurosurgery, Rhode Island Hospital/Warren Alpert Medical School of Brown University, Providence, USA

**Keywords:** anaplastic variant diffuse large b-cell lymphoma, cd30, cd10, bcl-6, mum1, germinal center b-cell, non-germinal center b-cell

## Abstract

Introduction

Diffuse large B-cell lymphoma (DLBCL) is an aggressive B-cell lymphoma and is the most common type of non-Hodgkin's lymphoma (NHL) worldwide. The World Health Organization (WHO) classification of hematopoietic tumors has recognized three morphological variants of DLBCL: centroblastic, immunoblastic, and anaplastic. Some studies have shown that the anaplastic variant of DLBCL is associated with aggressive clinicopathological features. Anaplastic DLBCL is rare, and the clinicopathological characteristics of this subtype of DLBCL are not widely studied in our population. Therefore, in this study, we evaluated the frequency of the anaplastic variant of DLBCL and its association with other clinicopathological parameters.

Methods

A retrospective study was conducted in the Department of Histopathology at the Liaquat National Hospital and Medical College over a period of six years, from January 2015 to December 2020. All cases diagnosed as DLBCL based on morphology and immunohistochemical (IHC) profile were included in the study. The diagnosis of anaplastic DLBCL was rendered based on morphology (large bizarre pleomorphic cells in a cohesive or sheet-like growth pattern), combined with CD30 IHC expression.

Results

The mean age of the patients was 52.90 ±16.42 years, and the mean Ki67 index was 73.18 ±16.52%. Of the 220 cases of DLBCL, 47.3% cases were germinal center B-cell (GCB) subtype, and 59.1% cases were nodal. BCL-2, BCL-6, MUM1, c-MYC, and CD10 positivity were noted in 60%, 45.5%, 40.9%, 44.1, and 38.6% cases, respectively. Only 14 cases (6.4%) were recognized as anaplastic variants of DLBCL according to the previously defined criterion. The only significant association of anaplastic-variant DLBCL was noted with a lack of BCL-2 expression. No significant association of anaplastic-variant DLBCL was noted with age, gender, Ki67 index, DLBCL subtype, or any other IHC marker expression.

Conclusion

We found a low frequency of the anaplastic variant of DLBCL in our study. No significant association of this DLBCL variant was noted with any of the clinicopathological parameters, except for the lack of BCL-2 expression. Alternatively, from a pathological perspective, it is important to recognize this variant of DLBCL as it often mimics other CD30-positive lymphoma and undifferentiated carcinoma.

## Introduction

Diffuse large B-cell lymphoma (DLBCL) is an aggressive B-cell lymphoma and is the most common kind of non-Hodgkin's lymphoma (NHL) worldwide [1,2]. With the advancement in molecular and gene-expression profiling studies, the spectrum of DLBCL has widened in the past few years and new subtypes have been widely accepted. The World Health Organization (WHO) classification of hematopoietic tumors has recognized three morphological variants of DLBCL: centroblastic, immunoblastic, and anaplastic. The differentiation between centroblastic and immunoblastic subtypes is often difficult and is associated with high inter-observer variability. Moreover, differentiating between centroblastic and immunoblastic subtypes of DLBCL is of limited prognostic significance. Alternatively, some studies have shown that the anaplastic variant of DLBCL is associated with aggressive clinicopathological features [3]. The anaplastic variant of DLBCL is characterized by larger pleomorphic and bizarre atypical lymphoid cells that may show a cohesive growth pattern resembling undifferentiated carcinoma or melanoma. Another characteristic feature of the anaplastic DLBCL is the diffuse expression of the CD30 immunohistochemical (IHC) marker. It is important to differentiate anaplastic DLBCL from anaplastic large cell lymphoma (ALCL), which is biologically and clinically an entirely different lymphoma. It is also vital to distinguish anaplastic DLBCL from rare lymphocyte-depleted Hodgkin's lymphoma (LD-HL). Anaplastic DLBCL is rare, and the clinicopathological characteristics of this subtype of DLBCL are not widely studied in our population. Therefore, in this study, we evaluated the frequency of anaplastic DLBCL and its association with other clinicopathological parameters.

## Materials and methods

**Figure 1:**
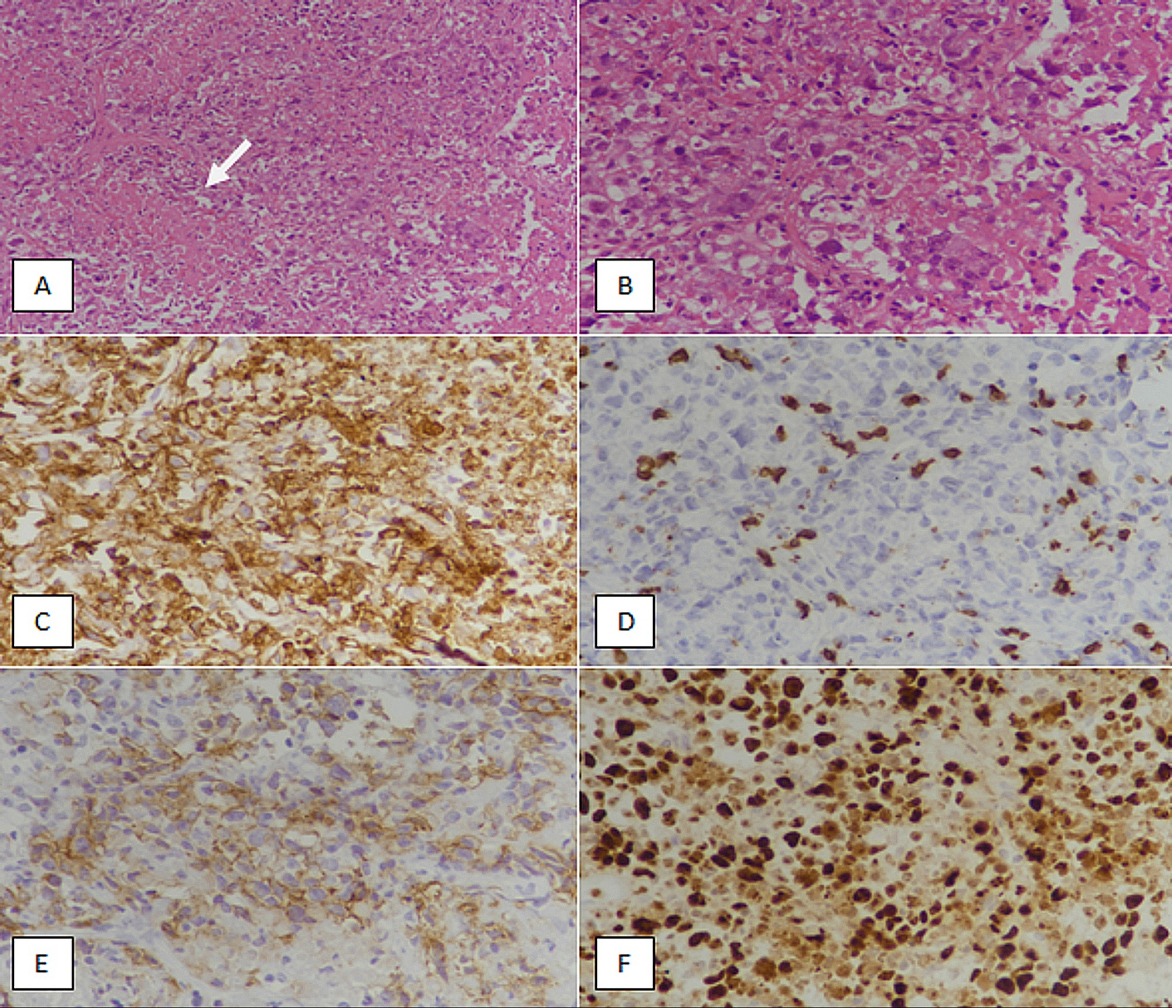
Anaplastic variant of diffuse large B-cell lymphoma (A): hematoxylin and eosin-stained section at 100x magnification showing malignant tumor mimicking an undifferentiated carcinoma. Areas of necrosis are evident (arrow). (B): hematoxylin and eosin-stained section at 400x magnification depicting tumor cells with a cohesive growth pattern. Tumor cells are large with moderate eosinophilic cytoplasm and atypical nuclei. (C): CD20 immunostaining revealing diffuse positivity in tumor cells. (D): CD3 immunomarker showing negative staining in tumor cells. Occasional reactive T-lymphocytes are highlighted in the background. (E): CD30 immunohistochemical staining depicting a positive expression in tumor cells. (F): Ki67 immunomarker showing 85% proliferative index in viable tumor cells

A retrospective study was conducted in the Department of Histopathology at the Liaquat National Hospital and Medical College over a period of six years, from January 2015 till December 2020. All cases diagnosed as DLBCL based on morphology and IHC profile were included in the study. All slides were retrieved and re-examined for confirmation of diagnosis. For the diagnosis of DLCL, an IHC panel including CD20, CD3, PAX5, CD5, CD23, and LCA was done. Moreover, pancytokeratin and S100 stains were done to exclude carcinoma and melanoma, respectively. The diagnosis was made in the light of light microscopic features and IHC studies by experienced histopathologists. Furthermore, IHC stains CD10, BCL-6, and MUM1 were performed to sub-categorize DLBCL into germinal cell B-cell (GCB) and non-germinal center B-cell (non-GCB) subtypes according to Han’s algorithm. A greater than 30% staining for CD10, MUM1, and BCL-6 was considered positive. Immunophenotypes including CD10+/any MUM1 and BCL-6, and CD10-/BCL-6+/MUM1- were labeled as GCB-subtype DLBCL. Alternatively, CD10-/MUM1+/BCL-6+, CD10-/MUM1+/BCL-6-, and CD10-/MUM1-/BCL-6- were considered non-GCB-subtype DLBCL. IHC markers BCL-2 and c-MYC were applied to distinguish double-expressor DLBCL from non-double-expressor DLBCL. Co-expression of greater than 40% c-MYC and greater than 50% BCL-2 was labeled as double-expressor DLBCL. Ki67 immunomarker was applied to reveal the proliferative index of tumor cells. Ki67 index was interpreted in the hot spots (highest number of staining cells) of the tumor and reported as an average percentage. CD30 IHC stain was applied to identify the anaplastic variant of DLBCL. The diagnosis of anaplastic DLBCL was rendered based on morphology (large bizarre pleomorphic cells in a cohesive or sheet-like growth pattern), combined with CD30 IHC expression (Figure 1).

Data analysis was performed using SPSS Statistics version 26.0 (IBM Inc., Armonk, NY). Independent t-test, chi-square test, and Fisher’s exact test were used to check the association. P-values of <0.05 were considered statistically significant.

## Results

The mean age of the patients was 52.90 ±16.42 years and most patients were above 50 years. The mean Ki67 index was 73.18 ±16.52%. Of the 220 cases of DLBCL, 47.3% cases were GCB subtype, and 59.1% cases were nodal. BCL-2, BCL-6, MUM1, c-MYC, and CD10 positivity were noted in 60%, 45.5%, 40.9%, 44.1, and 38.6% cases, respectively. Only 14 cases (6.4%) were recognized as anaplastic variants of DLBCL according to the previously defined criterion (Table 1).

**Table 1:** Descriptive statistics of the study population (n=220) DLBCL: diffuse large B-cell lymphoma; SD: standard deviation

Clinicopathological characteristics and immunohistochemical expression	Values
Age (years), mean ±SD	52.90 ±16.42
Ki67 (%), mean ±SD	73.18 ±16.52
Age groups	
≤35 years, n (%)	38 (17.3)
36-50 years, n (%)	45 (20.5)
>50 years, n (%)	137 (62.3)
Gender	
Male, n (%)	123 (55.9)
Female, n (%)	97 (44.1)
Subtype of DLBCL	
Germinal center B-cell subtype, n (%)	104 (47.3)
Non-germinal center B-cell subtype, n (%)	116 (52.7)
Site	
Nodal, n (%)	130 (59.1)
Extra-nodal, n (%)	90 (40.9)
Specimen type	
Trucut biopsy, n (%)	102 (46.4)
Excision biopsy, n (%)	118 (53.6)
BCL-2	
Positive, n (%)	132 (60)
Negative, n (%)	88 (40)
BCL-6	
Positive, n (%)	100 (45.5)
Negative, n (%)	120 (54.5)
MUM1	
Positive, n (%)	90 (40.9)
Negative, n (%)	130 (59.1)
c-MYC	
Positive, n (%)	97 (44.1)
Negative, n (%)	123 (55.9)
CD10	
Positive, n (%)	85 (38.6)
Negative, n (%)	135 (61.4)
Anaplastic variant of DLBCL	
Yes, n (%)	14 (6.4)
No, n (%)	206 (93.6)

Table 2 shows the association of clinicopathological characteristics of patients with the anaplastic variant of DLBCL. The only significant association of anaplastic variant DLBCL was noted with a lack of BCL-2 expression. No significant association of anaplastic variant DLBCL was noted with age, gender, Ki67 index, DLBCL subtype, or any other IHC marker expression.

**Table 2:** Association of the anaplastic variant of diffuse large B-cell lymphoma with clinicopathological characteristics *Independent t-test was applied; **Fisher’s exact test was applied; ***Chi-square test was applied; ****p-value significant as <0.05 DLBCL: diffuse large B-cell lymphoma; SD: standard deviation

Clinicopathological characteristics and immunohistochemical expression	Values		P-value
	Anaplastic variant of DLBCL		
	Yes	No	
Age (years)*, mean ±SD	45.28 ±22.06	53.41 ±15.90	0.197
Ki67 (%)*, mean ±SD	68.57 ±11.98	73.50 ±16.76	0.281
Age groups**			
≤35 years, n (%)	4 (28.6)	34 (16.5)	0.491
36-50 years, n (%)	2 (14.3)	43 (20.9)	
>50 years, n (%)	8 (57.1)	129 (62.6)	
Gender***			
Male, n (%)	4 (28.6)	119 (57.8)	0.033*
Female, n (%)	10 (71.4)	87 (42.2)	
Subtype of DLBCL***			
Germinal center B-cell subtype, n (%)	6 (42.9)	98 (47.6)	0.732
Non-germinal center B-cell subtype, n (%)	8 (57.1)	108 (52.4)	
Site***			
Nodal, n (%)	10 (71.4)	120 (58.3)	0.332
Extra-nodal, n (%)	4 (28.6)	86 (41.7)	
BCL-2***			
Positive, n (%)	4 (28.6)	128 (62.1)	0.013****
Negative, n (%)	10 (71.4)	78 (37.9)	
BCL-6***			
Positive, n (%)	8 (57.1)	92 (44.7)	0.364
Negative, n (%)	6 (42.9)	114 (55.3)	
MUM1***			
Positive, n (%)	4 (28.6)	86 (41.7)	0.332
Negative, n (%)	10 (71.4)	120 (58.3)	
c-MYC***			
Positive, n (%)	6 (42.9)	91 (44.2)	0.923
Negative, n (%)	8 (57.1)	115 (55.8)	
CD10***			
Positive, n (%)	4 (28.6)	81 (39.3)	0.424
Negative, n (%)	10 (71.4)	125 (60.7)	

## Discussion

Based on our findings, anaplastic DLBCL is a rare variant of DLBCL. We did not find any significant association of the anaplastic variant with clinicopathological features, except for its association with a lack of BCl-2 expression.

Some authors have suggested that anaplastic DLBCL is an aggressive subtype of DLBCL. Li et al. studied various clinicopathological aspects of anaplastic DLBCL and found that 86% of the cases were associated with a non-GCB immunophenotype and 51% of the cases had CD30 expression along with positive p53 staining in 80% of cases [3]. An overall poor survival (16 months) was reported, and 43% of the cases were double-expressor (i.e., expressed both BCL-2 and c-MYC). Genetic studies were significant in that 91% of the cases were positive for RELA, RELB, or c-Rel, and thus indicated the activation of the NFκB signaling pathway [3].

Sakakibara et al. reported a series of two anaplastic DLBCL cases having a cellular appearance similar to ALCL’s hallmark cells and recommended distinguishing them by performing immunohistochemistry for CD20, CD79a, CD30, and anaplastic lymphoma kinase (ALK) [4]. Kos et al. reported an anaplastic DLBCL case presenting as a left hilar mass with bilateral hilar and mediastinal lymphadenopathy and the large bizarre cells expressing CD20, PAX5, CD30, and MUM1 [5].

Asano et al. reported a case of anaplastic DLBCL involving the cervical lymph node with cutaneous involvement. The tumor was positive for CD30, CD45, and PAX5, but negative for CD10, CD20, CD3, CD15, BCL-2, Epstein-Barr virus-encoded small RNA (EBER), and ALK, and the patient showed an excellent response to cyclophosphamide, doxorubicin hydrochloride (hydroxydaunorubicin), vincristine sulfate (oncovin), and prednisone (CHOP) regimen chemotherapy and achieved complete remission [6]. More studies are required to investigate and assess clinicopathologic parameters and genetic changes associated with anaplastic DLBCL. Some earlier studies have also mentioned the strong CD30 expression in this subtype of DLBCL and emphasized the need for further studies [7].

Some authors have highlighted the distinctive histological features of the nodal anaplastic variant of DLBCL in contrast to extra-nodal cases. Megahed et al., in a study involving 31 cases of the anaplastic variant of DLBCL, showed that nodal involvement of the anaplastic variant of DLBCL was characterized by a distinctive sinusoidal pattern [8]. Such a distinctive nodal involvement was not noted in our cases.

Due to the presence of highly pleomorphic tumor cells, carcinoma and melanoma need to be excluded before making a diagnosis of anaplastic DLBCL. This distinction can be easily done with the help of pancytokeratin and S100/HMB45 IHC stains as anaplastic DLBCL is negative for these markers. Secondly, owing to CD30 positivity, embryonal carcinoma (EC), LD-HL, and ALCL are the differential diagnoses of anaplastic DLBCL. EC can easily be excluded as it is OCT3/4- and CKAE1/AE3-positive (anaplastic DLBCL is negative) and LCA- and CD20-negative (anaplastic DLBCL is positive). Distinguishing anaplastic DLBCL from LD-HL and ALCL is sometimes difficult; however, ALCL is negative for PAX5 and CD20, and LD-HL is also negative for CD20 and LCA, while the opposite is true for anaplastic DLBCL. Moreover, LD-HL is positive for CD15, while anaplastic DLBCL is negative.

The limitations of our study include its retrospective design and a lack of availability of molecular studies to identify genetic rearrangements characteristic of DLBCL. Moreover, clinical follow-up data were not available to compare survival differences in anaplastic DLBCL with those of other DLBCL subtypes.

## Conclusions

Anaplastic DLBCL is a rare morphological subtype of DLBCL characterized by atypical bizarre tumor histology along with diffuse CD30 expression. Although we did not find any significant association of anaplastic DLBCL with any clinicopathological parameters, future studies are warranted to identify any differences in disease-specific survival. Additionally, it is important to differentiate anaplastic DLBCL from its morphological mimickers such as undifferentiated carcinoma, melanoma, and especially LD-HL and ALCL, owing to the diffuse CD30 expression.
